# Quercetin–Arctigenin Co-Treatment Induces Mitochondrial Dysfunction and Apoptotic Cell Death Through Metabolic Stress in Malignant Mesothelioma Cells

**DOI:** 10.3390/life16050774

**Published:** 2026-05-06

**Authors:** Moon-Kyun Cho, Sang-Han Lee, Hae-Seon Nam, Yoon-Jin Lee

**Affiliations:** 1 Division of Molecular Cancer Research, Soonchunhyang Medical Research Institute, Soonchunhyang University, Cheonan 31511, Republic of Korea; mkcho@schmc.ac.kr (M.-K.C.); m1037624@sch.ac.kr (S.-H.L.); namhs@sch.ac.kr (H.-S.N.); 2 Department of Dermatology, Soonchunhyang University Hospital, Seoul 04401, Republic of Korea; 3 Department of Biochemistry, College of Medicine, Soonchunhyang University, Cheonan 31511, Republic of Korea; 4 Department of Tropical Medicine, College of Medicine, Soonchunhyang University, Cheonan 31511, Republic of Korea

**Keywords:** malignant mesothelioma, quercetin, arctigenin, metabolic stress, mitochondrial regulation, AMPK activation, apoptosis, polyphenols

## Abstract

Malignant mesothelioma is an aggressive cancer with limited therapeutic options, highlighting the need for novel strategies targeting metabolic vulnerabilities. Natural polyphenols have gained attention due to their ability to modulate cellular metabolism and apoptosis-related signaling pathways. In this study, we investigated the combined anticancer effects of quercetin (QUE) and arctigenin (ATG) in human malignant mesothelioma cells. QUE and ATG reduced the viability of MSTO-211H cells in a time-dependent manner, while non-malignant mesothelial MeT-5A cells showed relatively limited sensitivity under the tested conditions. Compared with single treatment, the combination treatment further enhanced growth inhibition, with combination index analysis suggesting a potential synergistic interaction. Co-treatment significantly decreased intracellular ATP levels and increased caspase-3/7 activity, suggesting metabolic stress-associated apoptotic responses. Annexin V analysis confirmed increased apoptotic cell populations following combination treatment. Western blot analysis demonstrated reduced expression of anti-apoptotic proteins Mcl-1 and Bcl-xL, along with increased cleavage of caspase-3 and PARP, consistent with involvement of intrinsic apoptosis-associated signaling pathways. In addition, increased phosphorylation of AMPK and altered expression of mitochondrial oxidative phosphorylation (OXPHOS) complex proteins were associated with potential alterations in mitochondrial respiratory protein expression. Collectively, these findings suggest that QUE and ATG co-treatment is associated with increased apoptotic cell death in malignant mesothelioma cells in association with metabolic stress–related mitochondrial functional alterations.

## 1. Introduction

Malignant mesothelioma is a highly aggressive tumor arising from mesothelial cells lining the pleural, peritoneal, or pericardial cavities and is closely linked to occupational or environmental exposure to asbestos fibers [1,2]. The disease is characterized by a prolonged latency period and is frequently diagnosed at advanced stages, contributing to poor clinical outcomes and limited therapeutic options [3,4,5]. Despite advances in systemic chemotherapy, immune checkpoint inhibitors, and multimodal treatment approaches, the overall survival of mesothelioma patients remains unsatisfactory [2,5]. Therapeutic resistance and biological heterogeneity further complicate treatment strategies, highlighting the need for novel approaches that target fundamental cellular vulnerabilities associated with tumor progression [4,5].

Reprogramming of cellular metabolism is widely recognized as a hallmark of cancer, supporting sustained proliferation, survival capacity, and adaptation to diverse cellular stress conditions [6,7]. Tumor cells frequently exhibit altered energy metabolism to maintain ATP production and redox homeostasis under conditions of hypoxia, nutrient limitation, and therapeutic pressure [7,8]. Mitochondria play a central role in these adaptive responses by regulating OXPHOS, reactive oxygen species generation, and apoptosis-associated signaling pathways [8,9]. Alterations in mitochondrial function have been associated with cancer progression, therapy resistance, and evasion of programmed cell death [9,10]. Targeting mitochondrial bioenergetics and metabolic plasticity has therefore emerged as a promising strategy for cancer therapy [6,10].

Naturally occurring polyphenolic compounds have attracted considerable interest due to their ability to influence molecular signaling pathways involved in cancer development and progression [11,12]. Polyphenols are widely distributed in plant-derived foods and, in various experimental systems, have been reported to exhibit anti-inflammatory, antioxidant, and anti-proliferative activities [11,13]. In addition to their effects on oxidative stress regulation, polyphenols may influence mitochondrial function, cellular metabolism, and apoptosis-associated signaling pathways [13,14]. Because many polyphenols demonstrate relatively low toxicity toward non-malignant cells, these compounds have been considered promising candidates for complementary therapeutic strategies aimed at improving selectivity while minimizing adverse effects [11,14].

Quercetin (QUE) is a naturally occurring flavonoid abundant in fruits and vegetables and has been extensively investigated for its potential biological activities in cancer-related experimental models [15,16,17]. Previous studies suggest that QUE may influence cell cycle progression, mitochondrial function, and apoptosis-associated signaling pathways [16,17]. Quercetin has also been reported to modulate intracellular ATP production and metabolic enzyme activity, indicating potential involvement in the regulation of cellular bioenergetics [17,18]. Furthermore, QUE has been associated with modulation of kinase-mediated signaling pathways related to cellular stress responses, suggesting possible relevance to the regulation of cancer cell survival mechanisms [18].

Arctigenin (ATG) is a lignan-derived polyphenolic compound isolated from plants such as *Arctium lappa* and has been investigated for biological activities related to metabolic regulation and cellular stress adaptation [19,20,21]. Previous studies suggest that ATG may influence mitochondrial function and energy metabolism under cellular stress conditions [19,20]. Arctigenin has also been reported to regulate apoptosis-associated signaling pathways and affect cancer cell survival through modulation of metabolic processes [20,21]. These observations indicate that ATG may contribute to the regulation of cellular stress responses associated with cancer progression.

Although QUE and ATG have individually been associated with anti-proliferative and pro-apoptotic effects in cancer cells, limited information is available regarding their combined effects in malignant mesothelioma. Because bioactive polyphenols may exert complementary regulatory actions on cellular metabolism and stress-associated signaling pathways, combination treatment strategies may enhance biological responses beyond those observed with individual compounds [14,18,21]. Cooperative modulation of mitochondrial function and apoptosis-related signaling pathways may therefore increase the susceptibility of cancer cells to metabolic stress conditions.

Cellular energy stress is frequently associated with decreased ATP production and activation of AMP-activated protein kinase (AMPK), a central regulator of cellular energy homeostasis [22]. AMPK functions as an intracellular energy sensor activated under conditions of metabolic imbalance and contributes to the regulation of mitochondrial activity, metabolic adaptation, and survival signaling pathways [22]. Activation of AMPK has been associated with inhibition of anabolic processes and induction of apoptosis under sustained metabolic stress conditions [22]. Because mitochondrial oxidative phosphorylation represents a major source of ATP production, modulation of mitochondrial respiratory activity may influence the susceptibility of cancer cells to apoptosis through regulation of cellular bioenergetic capacity [9,22].

**Figure 1:**
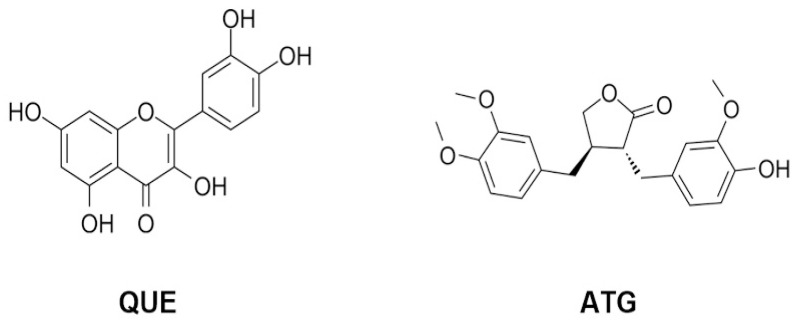
Chemical structures of the polyphenolic compounds quercetin (QUE) and arctigenin (ATG) were evaluated in this study.

In the present study, we investigated whether combined treatment with QUE and ATG could influence cell viability, apoptosis induction, and mitochondrial stress-associated signaling pathways in malignant mesothelioma cells. Experimental concentrations were selected based on conditions maintaining greater than 95% viability in non-malignant mesothelial MeT-5A cells, allowing evaluation of differential responses under the tested conditions. Figure 1 presents the chemical structures of QUE and ATG. The effects of single and combined treatment were evaluated through analysis of cell viability, intracellular ATP levels, apoptosis-associated markers, and mitochondrial regulatory proteins. These findings provide mechanistic insight into cooperative regulatory effects of polyphenols on cellular stress responses associated with mesothelioma cell survival and may contribute to the development of therapeutic strategies targeting cancer-associated metabolic vulnerabilities.

## 2. Materials and Methods

### 2.1. Chemicals and Reagents

Quercetin (QUE) and arctigenin (ATG) were obtained from Sigma-Aldrich (St. Louis, MO, USA). Stock solutions were prepared in dimethyl sulfoxide (DMSO; Sigma-Aldrich), then diluted in culture medium immediately prior to use. The final concentration of DMSO did not exceed 0.1% (*v*/*v*) in any experimental condition. Primary antibodies against Mcl-1 (cat. no. 5453), Bcl-xL (cat. no. 2764), Bcl-2 (cat. no. 2870), cleaved caspase-3 (cat. no. 9664), cleaved PARP (cat. no. 5625), AMPK (cat. no. 2532), and phospho-AMPK (Thr172; cat. no. 2535) were obtained from Cell Signaling Technology (Danvers, MA, USA). The Total OXPHOS Human WB Antibody Cocktail (cat. no. 45-8199) was purchased from Thermo Fisher Scientific (Waltham, MA, USA). β-actin antibody (cat. no. A2228) was obtained from Sigma–Aldrich (St. Louis, MO, USA). Horseradish peroxidase (HRP)-conjugated secondary antibodies were obtained from Santa Cruz Biotechnology (Dallas, TX, USA). MTT reagent, crystal violet, and other analytical-grade reagents were obtained from Sigma-Aldrich, unless otherwise specified.

### 2.2. Cell Culture

Human malignant mesothelioma MSTO-211H cells and human mesothelial MeT-5A cells were obtained from the American Type Culture Collection (ATCC, Manassas, VA, USA). Cells were maintained in a humidified incubator at 37 °C in an atmosphere containing 5% CO_2_. MSTO-211H cells were maintained in Dulbecco’s Modified Eagle Medium (DMEM; Welgene Inc., Gyeongsan, Republic of Korea) supplemented with 5% fetal bovine serum (FBS; Gibco, Gaithersburg, MD, USA) and 1% penicillin–streptomycin (Welgene Inc., Gyeongsan, Republic of Korea). MeT-5A cells were cultured in Medium 199 supplemented with 10% FBS and growth supplements, according to the supplier’s recommendations. Cells were passaged using 0.25% trypsin–EDTA (Welgene) and, to ensure phenotypic stability, were used within a limited passage number.

### 2.3. Cell Viability Assay

Cell viability was assessed using an MTT-based colorimetric assay. Cells were seeded in 96-well plates at a density of 1 × 10^4^ cells per well and allowed to attach for 24 h prior to treatment. Cells were treated with QUE, ATG, or a combination of QUE and ATG for the indicated time periods. Following treatment, MTT solution (Sigma-Aldrich, St. Louis, MO, USA; final concentration of 0.1 mg/mL) was added and incubated for 2–3 h at 37 °C. Formazan crystals were dissolved in DMSO, and absorbance was measured at 550 nm using a GloMax^®^ Multi Detection System (Promega, Madison, WI, USA). Cell viability was expressed as a percentage relative to untreated control cells. Because the MTT assay reflects mitochondrial metabolic activity, the measured values represent relative metabolic activity associated with viable cells.

### 2.4. Combination Analysis

The interaction between QUE and ATG was assessed using the combination index (CI) method based on the Chou–Talalay principle. CI values were calculated using CompuSyn software version 1.0 (ComboSyn Inc., Paramus, NJ, USA). CI values < 1 indicate synergistic interaction, CI = 1 indicates an additive effect, and CI > 1 indicates antagonistic interaction.

### 2.5. ATP Measurement

Intracellular ATP levels were determined using a luminescence-based ATP detection assay (CellTiter-Glo^®^ 2.0, Promega, Madison, WI, USA), according to the manufacturer’s protocol. Following treatment with QUE, ATG, or QUE + ATG, ATP detection reagent was added directly to the culture wells, and luminescence intensity was measured using a GloMax^®^ Multi microplate reader (Promega, Madison, WI, USA). ATP levels were normalized to untreated control cells under identical experimental conditions.

### 2.6. Crystal Violet Staining

Cell survival was further evaluated using crystal violet staining. Cells were seeded into 6–well plates, and treated with the indicated compounds for 48 h. After treatment, cells were fixed in 100% methanol for 15 min, and stained with 0.5% crystal violet solution for 20 min at room temperature (RT). Excess stain was removed by washing with distilled water, and stained cells were air-dried. Images were obtained using a microscope, and the bound dye was solubilized in 10% acetic acid. Absorbance was measured at 570 nm using a microplate reader.

### 2.7. Apoptosis Analysis

Apoptotic cell populations were quantified using the Muse™ Annexin V & Dead Cell Assay kit according to the manufacturer’s guidelines (cat. no. MCH100105; Merck Millipore, Darmstadt, Germany), following the manufacturer’s protocol. After treatment, cells were harvested, washed with phosphate-buffered saline (PBS), and resuspended in assay buffer. Cells were incubated with Annexin V reagent for 20 min at RT in the dark. Samples were analyzed using a Muse^™^ Cell Analyzer (Merck Millipore, Daemstadt, Germany), and the percentages of viable, early apoptotic, late apoptotic, and dead cells were calculated.

### 2.8. Western Blot Analysis

Protein expression levels were analyzed by Western blotting. Cells were lysed using RIPA buffer (Thermo Fisher Scientific, Waltham, MA, USA) supplemented with protease and phosphatase inhibitor cocktails (Roche Diagnostics, Basel, Switzerland). Protein concentration was determined using a bicinchoninic acid (BCA) protein assay kit (cat. no. 23225; Thermo Fisher Scientific). Equal amounts of protein (40 µg) were separated using NuPAGE™ 4–12% Bis–Tris gradient gels (Invitrogen, Carlsbad, CA, USA) and transferred onto polyvinylidene difluoride (PVDF) membranes (Millipore). Membranes were blocked with 5% skim milk or protein blocking buffer for 1 h at RT and incubated with primary antibodies overnight at 4 °C. After incubation with HRP-conjugated secondary antibodies, immunoreactive bands were visualized using enhanced chemiluminescence (ECL) detection reagents (Cytiva, Marlborough, MA, USA). Signals were detected using X-ray film, and band intensities were quantified using ImageJ software (version 1.54; National Institutes of Health, Bethesda, MD, USA). Band intensities were normalized to β-actin for each corresponding lane. Representative images from independent experiments are shown.

### 2.9. Statistical Analysis

All experiments were independently performed at least three times. Data are presented as the mean ± standard deviation (SD). Statistical comparisons between groups were performed using one-way analysis of variance (ANOVA), followed by Tukey’s post hoc test using GraphPad Prism software (version 9.5.1; GraphPad Software Inc., San Diego, CA, USA). The number of independent biological replicates (n) is indicated in the corresponding figure legends.

## 3. Results

### 3.1. Determination of Non-Cytotoxic Concentrations of Quercetin and Arctigenin Based on MTT Analysis

**Figure 2:**
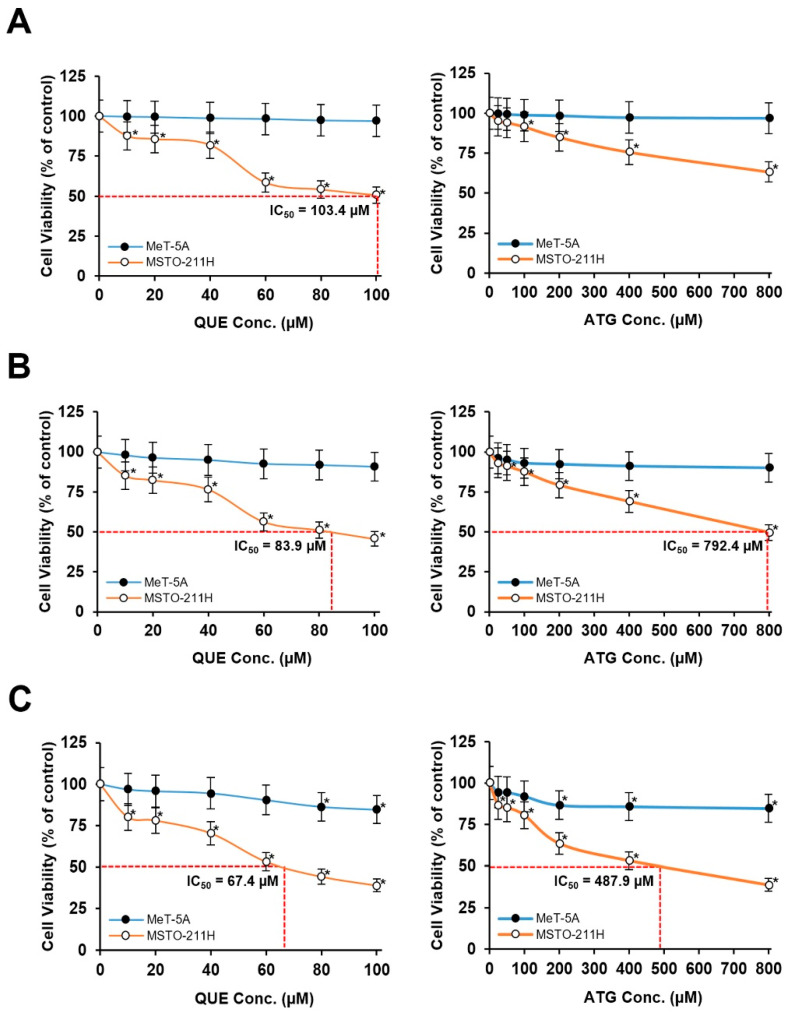
Effects of quercetin and arctigenin on relative metabolic activity in mesothelioma and mesothelial cells. (**A**–**C**) MSTO-211H and MeT-5A cells were treated with increasing concentrations of QUE or ATG for (**A**) 24 h, (**B**) 48 h, and (**C**) 72 h. Relative metabolic activity was measured using the MTT assay. Data are presented as the mean ± SD from at least three independent experiments. Statistical significance was determined using one-way ANOVA followed by Tukey’s post hoc test (* *p* < 0.05 vs. control).

To determine appropriate experimental concentrations for combination treatment, the effects of QUE and ATG on cell viability were first evaluated in malignant mesothelioma MSTO-211H cells and non-malignant mesothelial MeT-5A cells using an MTT assay (Figure 2). Cells were treated with increasing concentrations of each compound for 24, 48, and 72 h to identify conditions that produce measurable effects in mesothelioma cells while minimizing changes in metabolic activity in MeT-5A cells.

QUE treatment resulted in a progressive reduction in metabolic activity of MSTO-211H cells in a time- and dose-dependent manner (Figure 2A–C). At 24 h, relatively modest changes were observed at concentrations up to 40 μM, whereas more pronounced reductions were detected at 48 and 72 h. Treatment with 40 μM QUE reduced the relative metabolic activity of MSTO-211H cells to approximately 80%, 65–70%, and 55–60% at 24, 48, and 72 h, respectively, compared with untreated control cells. Increasing concentrations of QUE further enhanced the observed effect, indicating increased sensitivity of mesothelioma cells to QUE exposure over time.

In contrast, MeT-5A cells exhibited minimal changes in metabolic activity under comparable treatment conditions. At concentrations up to 40 μM, MeT-5A metabolic activity remained above 95% at all examined time points, indicating limited effects on cellular metabolic activity under the tested conditions (Figure 2A–C). At higher concentrations, modest reductions were observed; however, the magnitude of change remained substantially smaller than that observed in MSTO-211H cells.

ATG treatment alone produced moderate reductions in relative metabolic activity in MSTO-211H cells (Figure 2A–C). Exposure to 50 μM ATG resulted in MSTO-211H metabolic activity values of approximately 88–92%, 75–80%, and 70% at 24, 48, and 72 h, respectively. These findings indicate that, compared with QUE treatment, ATG alone exerts a measurable but less pronounced effect under the same experimental conditions.

Importantly, following ATG exposure across the tested concentration range, MeT-5A cells maintained high metabolic activity. At 50 μM ATG, MeT-5A metabolic activity remained above 95% at 24, 48, and 72 h (Figure 2A–C), supporting the suitability of this concentration for combination experiments designed to minimize non-specific effects on non-malignant cells.

Based on these findings, concentrations that maintained greater than 95% metabolic activity in MeT-5A cells were selected for subsequent combination studies. Specifically, 40 μM QUE and 50 μM ATG were chosen as experimental concentrations because they produced measurable effects in MSTO-211H cells while preserving high metabolic activity in non-malignant mesothelial cells.

Among the tested time points, 48 h was selected as the primary condition for subsequent mechanistic analyses because this time point provided a balance between detectable effects in mesothelioma cells and minimal alterations in MeT-5A cells. At 24 h, changes in metabolic activity were relatively modest, whereas 72 h treatment produced more pronounced reductions that may reflect secondary cellular stress responses. In contrast, the 48 h condition allowed clear discrimination between malignant and non-malignant cells while maintaining MeT-5A metabolic activity above 95%.

Importantly, the selected concentrations fall within ranges commonly used in mechanistic in vitro studies of polyphenols, supporting evaluation of cellular responses under controlled experimental conditions while minimizing non-specific effects.

Therefore, these conditions provide an experimental framework suitable for investigating differential metabolic responses and apoptosis-associated signaling changes following combination treatment.

Collectively, these results demonstrate differential sensitivity between mesothelioma cells and non-malignant mesothelial cells to QUE and ATG exposure under the tested conditions. Selection of experimental concentrations based on preservation of MeT-5A metabolic activity above 95% provides a rationale for subsequent investigation of cooperative effects of QUE and ATG on mesothelioma cell survival and apoptosis-related signaling pathways.

### 3.2. Combined Treatment Reduces Cell Viability and Cell Growth Capacity in MSTO−211H Cells

**Figure 3:**
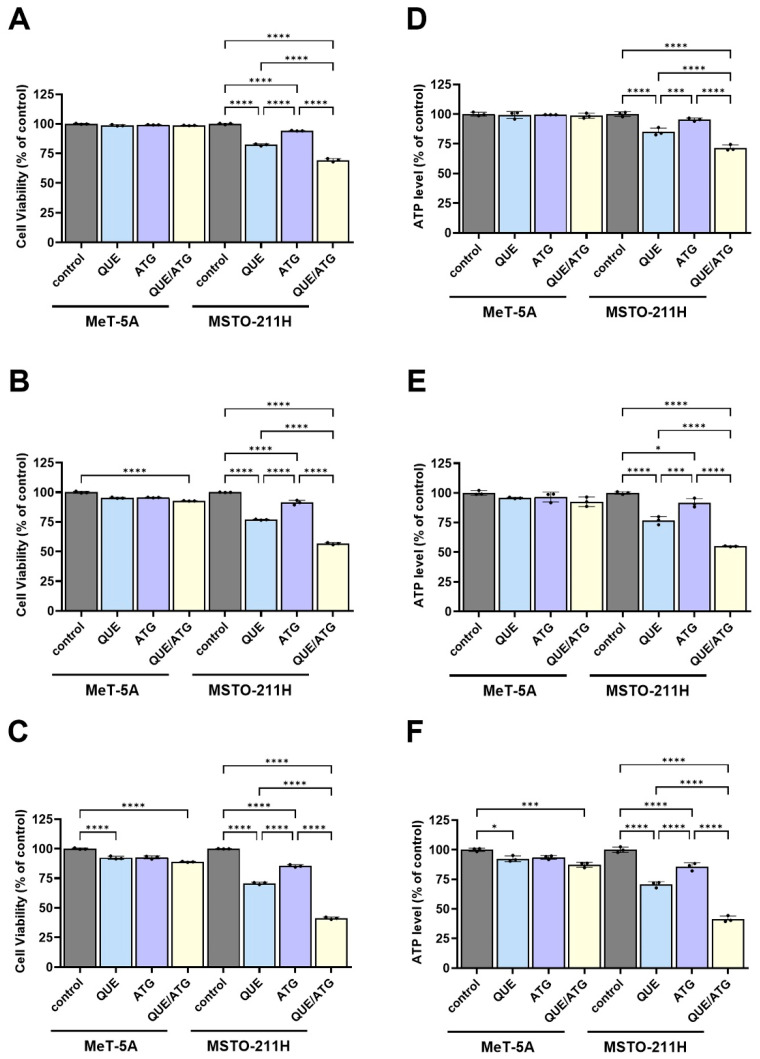
Combined treatment with quercetin and arctigenin reduces metabolic activity and intracellular ATP production in mesothelioma cells. (**A**–**C**) MSTO-211H and MeT-5A cells were treated with QUE (40 μM), ATG (50 μM), or their combination for (**A**) 24 h, (**B**) 48 h, and (**C**) 72 h. Relative metabolic activity was measured using the MTT assay. (**D**–**F**) Intracellular ATP levels were measured after (**D**) 24 h, (**E**) 48 h, and (**F**) 72 h treatment. Data are presented as the mean ± SD from at least three independent experiments. Statistical significance was determined using one-way ANOVA followed by Tukey’s post hoc test (* *p* < 0.05, *** *p* < 0.001, **** *p* < 0.0001 vs. control).

To determine whether combined treatment with QUE and ATG enhances inhibitory effects on mesothelioma cell growth, MSTO-211H and MeT-5A cells were treated with QUE (40 μM), ATG (50 μM), or their combination for 24, 48, and 72 h. Relative metabolic activity was assessed using the MTT assay, and intracellular ATP levels were evaluated to assess metabolic stress (Figure 3).

In MSTO-211H cells, treatment with QUE or ATG alone produced moderate reductions in relative metabolic activity across the examined time points (Figure 3A–C). Treatment with QUE alone decreased relative metabolic activity to approximately 80%, 65–70%, and 55–60% at 24, 48, and 72 h, respectively. ATG treatment alone resulted in slightly weaker effects, with relative metabolic activity remaining at approximately 88–92%, 75–80%, and 70% at 24, 48, and 72 h, respectively. These findings indicate that, when administered individually, both compounds exert inhibitory effects on mesothelioma cell metabolic activity.

Notably, combined treatment with QUE and ATG produced a greater reduction in MSTO-211H relative metabolic activity compared with either compound alone. Co-treatment decreased relative metabolic activity relative to untreated control cells to approximately 70–75%, 58–62%, and 40–48% at 24, 48, and 72 h, respectively. The reduction observed in the combination group was consistently greater than that observed in single-treatment groups at all examined time points, indicating enhanced inhibitory effects under co-treatment conditions.

In contrast, MeT-5A cells showed minimal changes in relative metabolic activity following treatment with QUE, ATG, or the combined treatment (Figure 3A–C). Relative metabolic activity remained above approximately 95% across all treatment conditions and time points, indicating limited effects in non-malignant mesothelial cells under the tested conditions. These findings support the relative selectivity of the combination treatment at the selected concentrations.

Measurement of intracellular ATP levels further supported the metabolic effects of combination treatment (Figure 3D–F). In MSTO-211H cells, combination treatment produced a more pronounced reduction in ATP levels than either single-treatment group. The decrease in intracellular ATP was observed at all examined time points and became more marked with longer exposure, indicating enhanced metabolic stress under co-treatment conditions. In contrast, MeT-5A cells showed relatively minor changes in ATP levels following treatment, consistent with the MTT assay results.

Overall, the greater inhibitory effect observed under combination treatment conditions suggests cooperative effects of QUE and ATG on cellular processes regulating mesothelioma cell metabolic activity and survival.

Taken together, these findings demonstrate that co-treatment with QUE and ATG enhances inhibitory effects on mesothelioma cell metabolic activity and intracellular ATP levels in a time-dependent manner while maintaining minimal effects in non-malignant mesothelial cells. The greater reduction observed in the combination group suggests that QUE and ATG exert complementary effects on cellular processes regulating mesothelioma cell metabolic activity, energy homeostasis, and survival.

### 3.3. Combined Treatment Enhances Apoptotic Cell Death

**Figure 4:**
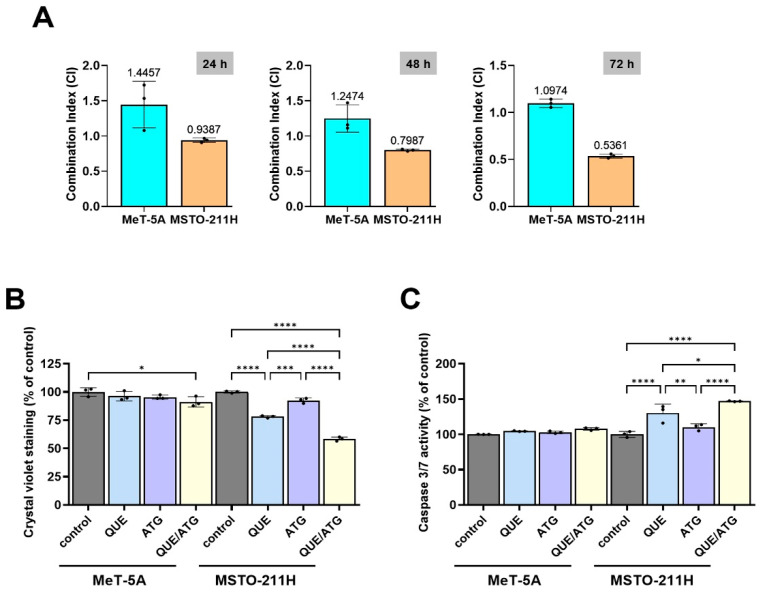
Combination treatment suggests a potential synergistic interaction, reduces cell growth capacity, and increases caspase activity in mesothelioma cells. (**A**) Combination index (CI) values were calculated for QUE (40 μM) and ATG (50 μM) co-treatment in MeT-5A and MSTO-211H cells at 24, 48, and 72 h. CI values < 1 may be indicative of a potential synergistic interaction under the experimental conditions tested. (**B**) Cell growth capacity was assessed by crystal violet staining following 48 h treatment with QUE, ATG, or their combination. (**C**) Caspase 3/7 activity was measured after 48 h of treatment to evaluate apoptosis-associated protease activation. Data are presented as the mean ± SD from at least three independent experiments. Statistical significance was determined using one-way ANOVA followed by Tukey’s post hoc test (* *p* < 0.05, ** *p* < 0.01, *** *p* < 0.001, **** *p* < 0.0001 vs. control).

To further evaluate whether cooperative interactions contribute to the observed reduction in relative metabolic activity following co-treatment with QUE and ATG, combination index (CI) analysis was performed to assess potential interactions between the two compounds (Figure 4A). CI values observed in MSTO-211H cells were below 1 at 48 h and 72 h, suggesting a potential synergistic interaction at later time points. In contrast, CI values in MeT-5A cells remained close to or above one, indicating limited interaction under non-malignant conditions. These findings support the interpretation that cooperative effects of QUE and ATG are more pronounced in mesothelioma cells.

Crystal violet staining was performed to evaluate long-term cell growth capacity following treatment (Figure 4B). In MSTO-211H cells, treatment with QUE or ATG alone produced moderate reductions in crystal violet staining intensity, indicating partial inhibition of cell growth capacity. Notably, combined treatment with QUE and ATG resulted in a greater decrease in crystal violet staining compared with either single-treatment condition. The reduction observed in the co-treatment group suggests enhanced suppression of cell growth capacity under combined exposure conditions. In contrast, MeT-5A cells showed relatively minor changes in crystal violet staining intensity across all treatment conditions, supporting limited effects of the selected concentrations in non-malignant mesothelial cells.

**Figure 5:**
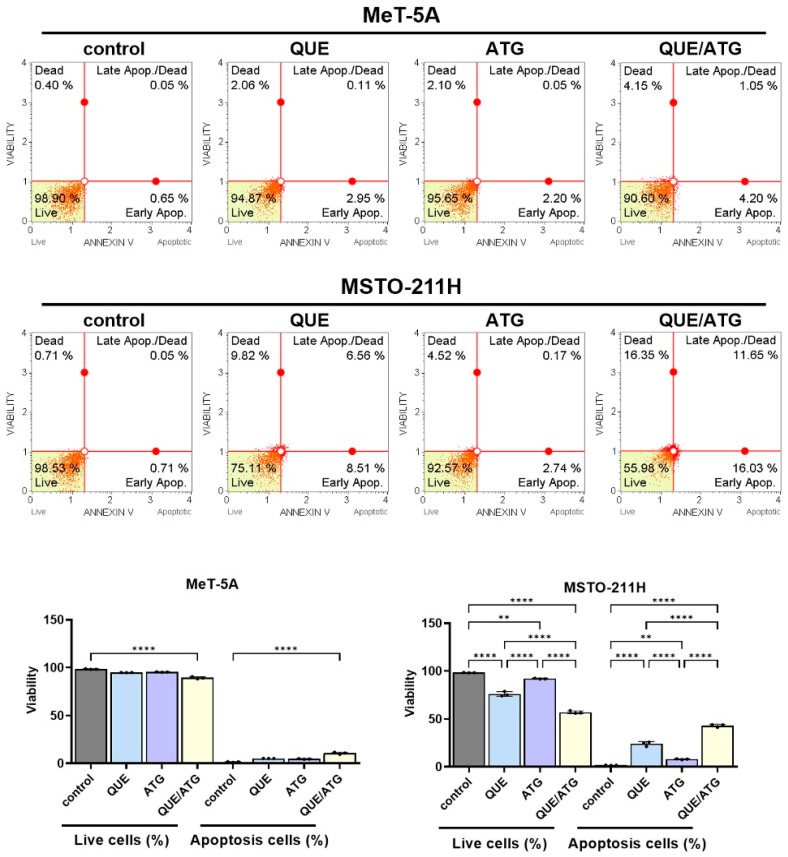
Combined treatment with quercetin and arctigenin suggests increased apoptotic cell populations in mesothelioma cells. Representative Annexin V dot plots showing apoptotic cell populations in MeT-5A and MSTO-211H cells following treatment with QUE (40 μM), ATG (50 μM), or their combination for 48 h. Dot plots represent cells stained with Annexin V and PI, where color intensity indicates cell density and quadrants correspond to live, early apoptotic, and late apoptotic cell populations. Percentages of live, early apoptotic, and late apoptotic cells are indicated. Lower panels show quantitative analysis of live cells (%) and total apoptotic cells (%). Data are presented as the mean ± SD from at least three independent experiments. Statistical significance was determined using one-way ANOVA followed by Tukey’s post hoc test (** *p* < 0.01, **** *p* < 0.0001 vs. control).

To determine whether the reduction in relative metabolic activity observed following co-treatment with QUE and ATG was associated with activation of apoptotic signaling pathways, the caspase activity and Annexin V staining analyses were performed in MSTO-211H cells (Figure 4C and Figure 5). These experiments were conducted to evaluate whether the observed metabolic alterations were accompanied by activation of apoptosis-associated signaling mechanisms.

Caspase activity analysis demonstrated increased activation of caspase-dependent proteolytic signaling following treatment with QUE and ATG (Figure 4C). Treatment with QUE (40 μM) alone resulted in a moderate increase in caspase activity of approximately 1.3–1.5-fold relative to untreated control cells. Similarly, ATG (50 μM) alone produced a modest increase in caspase activity, typically ranging approximately 1.2–1.4-fold compared with control levels. Importantly, combined treatment with QUE and ATG resulted in a greater increase in caspase activity, reaching approximately 1.8–2.2-fold relative to control cells. The greater activation of caspase 3/7 observed under co-treatment conditions suggests enhanced activation of apoptosis-associated protease activity compared with single-treatment conditions.

To further evaluate apoptotic cell death, Annexin V staining was performed to determine the proportion of apoptotic cells following treatment (Figure 5). In untreated MSTO-211H control cells, the proportion of Annexin V-positive cells remained relatively low, typically below approximately 8–10%. Treatment with QUE alone moderately increased the total apoptotic population to approximately 15–18%, indicating measurable induction of apoptosis. ATG treatment alone produced a comparable increase in apoptotic cells, typically reaching approximately 14–17% total apoptosis.

Notably, compared with single-treatment conditions, co-treatment with QUE and ATG resulted in a marked increase in apoptotic cell populations. Relative to control and single-agent treatment groups, combined treatment increased total apoptotic cell fractions to approximately 35–45%, representing a substantial increase. Following combined exposure to QUE and ATG, both early apoptotic and late apoptotic cell populations were increased, suggesting progression of apoptotic processes. Early apoptotic cells increased approximately two-fold relative to single-treatment groups, while late apoptotic populations also showed an increasing trend, indicating enhanced apoptotic progression under co-treatment conditions.

In contrast, following treatment with QUE, ATG, or their combination, MeT-5A cells exhibited relatively minor changes in apoptotic cell populations. The total Annexin V-positive population remained below approximately 10–12% across all treatment conditions, indicating minimal induction of apoptosis in non-malignant mesothelial cells. Similarly, caspase activity levels in MeT-5A cells showed only small variations between treatment groups, supporting selective responses of QUE and ATG in mesothelioma cells under the experimental conditions used in this study.

Overall, these findings support activation of caspase-dependent apoptotic signaling pathways in mesothelioma cells following combination treatment.

Taken together, these findings demonstrate that co-treatment with QUE and ATG enhances activation of caspase-dependent apoptotic signaling pathways and increases apoptotic cell populations in MSTO-211H mesothelioma cells. The increased caspase activity observed in Figure 4C is consistent with elevated Annexin V–positive populations observed in Figure 5, indicating that combined treatment promotes apoptosis-associated cellular responses. These findings are consistent with the anti-proliferative effects observed in previous experiments and suggest that cooperative modulation of apoptosis-associated signaling pathways may contribute to increased susceptibility of mesothelioma cells to combined QUE and ATG treatment.

### 3.4. Co-Treatment Regulates Mitochondrial Apoptosis-Related Signaling

**Figure 6:**
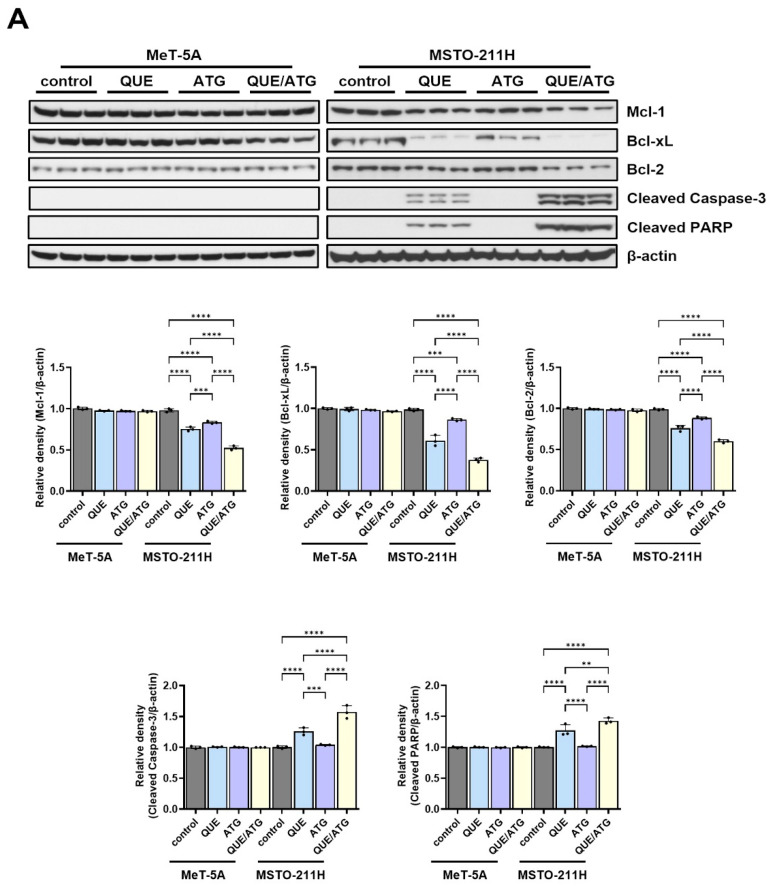

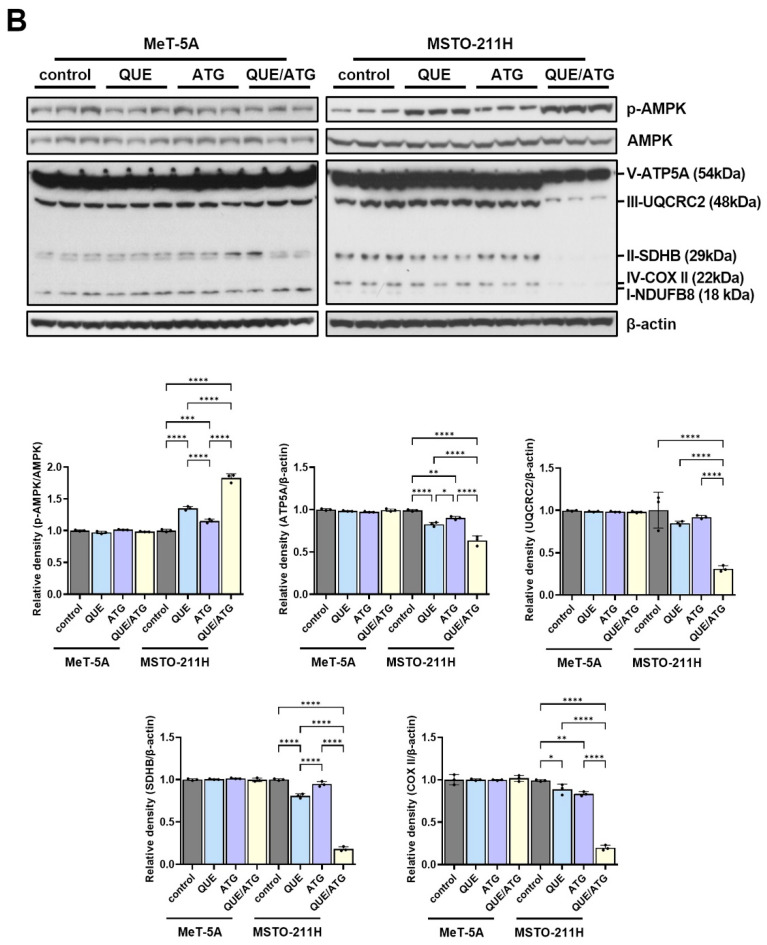
Co-treatment with quercetin and arctigenin is associated with modulation of mitochondrial apoptosis-associated signaling and energy stress-related protein expression in mesothelioma cells. (**A**) Representative Western blot analysis of apoptosis-associated proteins in MeT-5A and MSTO-211H cells treated with quercetin (QUE, 40 μM), arctigenin (ATG, 50 μM), or their combination for 48 h. Expression levels of anti-apoptotic proteins Mcl-1, Bcl-xL, and Bcl-2, as well as apoptosis execution markers cleaved caspase-3 and cleaved PARP, were examined. β-actin was used as the loading control. (**B**) Representative Western blot analysis of energy stress- and mitochondrial protein expression-related markers. Expression levels of phosphorylated AMPK (p-AMPK), total AMPK, and selected oxidative phosphorylation (OXPHOS) complex subunits (ATP5A, UQCRC2, SDHB, COX II, and NDUFB8) were evaluated in MeT-5A and MSTO-211H cells following treatment with QUE, ATG, or their combination for 48 h. Relative protein expression levels were normalized to β-actin, and the p-AMPK/AMPK ratio was calculated to evaluate AMPK activation. Densitometric values are presented as mean ± SD from three independent experiments (n = 3). Statistical significance was determined using one-way ANOVA followed by Tukey’s post hoc test (* *p* < 0.05, ** *p* < 0.01, *** *p* < 0.001, **** *p* < 0.0001 vs. control). Faint background signals may be present due to exposure sonditions and do not affect the interpretation of the results.

To further investigate the molecular mechanisms underlying the enhanced apoptotic response induced by co-treatment with quercetin (QUE) and arctigenin (ATG), the expression levels of mitochondrial apoptosis-associated proteins and bioenergetic regulators were analyzed by Western blotting (Figure 6). The analysis focused on key regulators of mitochondrial integrity, apoptosis signaling, and cellular energy metabolism, including anti-apoptotic proteins (Mcl-1, Bcl-2, Bcl-xL), apoptosis execution markers (cleaved caspase-3 and cleaved PARP), AMP-activated protein kinase (AMPK), and mitochondrial oxidative phosphorylation (OXPHOS) complex subunits.

Co-treatment with QUE and ATG resulted in a pronounced reduction in anti-apoptotic Bcl-2 family proteins in MSTO-211H mesothelioma cells compared with single-treatment conditions. Densitometric analysis showed that Mcl-1 expression decreased to 0.52-fold relative to control following combined treatment, whereas single treatment with QUE or ATG resulted in values of 0.75-fold and 0.83-fold, respectively. Similarly, Bcl-xL expression was markedly reduced to 0.38-fold in the co-treatment group, compared with 0.61-fold and 0.86-fold in QUE- and ATG-treated cells, respectively. Bcl-2 expression also showed a reduction to 0.60-fold following combined treatment, whereas single treatment resulted in values of 0.76-fold (QUE) and 0.88-fold (ATG). In contrast, expression of these anti-apoptotic proteins remained largely unchanged in MeT-5A cells (0.97–1.00-fold relative to control), indicating minimal changes in non-malignant mesothelial cells.

Consistent with activation of apoptosis-associated signaling pathways, increased expression of apoptosis execution markers was observed in MSTO-211H cells following co-treatment. Cleaved caspase-3 levels increased to 1.57-fold relative to control in the combination group, whereas single treatment resulted in values of 1.26-fold (QUE) and 1.04-fold (ATG). Similarly, cleaved PARP expression increased to 1.43-fold following co-treatment, compared with 1.27-fold and 1.02-fold in QUE- and ATG-treated cells, respectively. In MeT-5A cells, cleaved caspase-3 and cleaved PARP expression remained essentially unchanged (0.99–1.01-fold), further supporting selective modulation of apoptosis-associated signaling in mesothelioma cells.

Because mitochondrial bioenergetic alterations are frequently associated with cellular energy stress responses, expression of phosphorylated AMPK (p-AMPK) was also evaluated. Co-treatment with QUE and ATG increased AMPK phosphorylation in MSTO-211H cells, reaching 1.83-fold relative to control. Single treatment with QUE or ATG resulted in more moderate increases of 1.35-fold and 1.15-fold, respectively. In contrast, p-AMPK levels in MeT-5A cells remained relatively stable (0.97–1.01-fold), indicating limited activation of energy stress signaling pathways in non-malignant cells under the tested conditions.

To assess potential alterations in mitochondrial respiratory protein expression, representative subunits of the OXPHOS complexes were examined. Co-treatment resulted in consistent reductions in multiple OXPHOS proteins in MSTO-211H cells. Expression of ATP5A (Complex V) decreased to 0.63-fold relative to control, compared with 0.83-fold and 0.90-fold following QUE and ATG treatment alone. Similarly, UQCRC2 (Complex III) expression was markedly reduced to 0.31-fold in the co-treatment group, whereas single treatment resulted in values of 0.85-fold (QUE) and 0.92-fold (ATG). SDHB (Complex II) expression showed a pronounced reduction to 0.18-fold following co-treatment, compared with 0.81-fold and 0.95-fold following QUE and ATG treatment alone. COX II (Complex IV) levels were also strongly decreased to 0.20-fold in the combination group, whereas single treatment resulted in moderate reductions of 0.89-fold (QUE) and 0.83-fold (ATG). In contrast, expression of NDUFB8 (Complex I) remained relatively stable across treatment conditions in MSTO-211H cells, suggesting differential sensitivity of OXPHOS components.

Overall, coordinated reduction in multiple OXPHOS complex subunits suggests altered mitochondrial respiratory protein expression and potential disruption of cellular bioenergetic homeostasis under co-treatment conditions.

OXPHOS protein expression remained relatively stable in MeT-5A cells across all treatment groups, with expression values generally ranging from 0.97- to 1.02-fold relative to control. These findings indicate that combined treatment preferentially modulates mitochondrial protein expression patterns in mesothelioma cells while maintaining relative stability in non-malignant mesothelial cells.

Taken together, these results suggest that co-treatment with QUE and ATG is associated with coordinated modulation of apoptosis-associated signaling proteins, AMPK activation, and OXPHOS protein expression in mesothelioma cells. The observed reduction in anti-apoptotic protein expression, increased cleavage of apoptosis markers, enhanced AMPK phosphorylation, and altered expression of multiple OXPHOS complex subunits collectively suggest potential involvement of metabolic stress-associated mitochondrial regulatory responses following combined exposure to QUE and ATG.

These findings support an association between metabolic stress-related signaling changes and apoptosis-associated cellular responses under the experimental conditions used in this study.

## 4. Discussion

This study demonstrates that combined treatment with quercetin and arctigenin enhanced inhibitory effects on mesothelioma cell viability while maintaining minimal cytotoxicity in non-malignant mesothelial MeT-5A cells. Experimental concentrations were selected based on preservation of MeT-5A viability above 95%, enabling evaluation of cancer-selective responses under conditions minimizing non-specific toxicity. This approach allows interpretation of the observed effects within the context of differential cellular susceptibility, which is particularly relevant when investigating potential therapeutic strategies targeting cancer-associated metabolic vulnerabilities.

Polyphenolic compounds have been widely studied for their capacity to modulate intracellular pathways associated with oxidative stress, inflammation, metabolic regulation, and programmed cell death [23,24,25]. Increasing evidence indicates that cancer cells exhibit altered metabolic requirements that support rapid proliferation and adaptation to stressful microenvironmental conditions [26,27,28]. These adaptations frequently involve changes in mitochondrial function, redox balance, and ATP production, allowing cancer cells to maintain bioenergetic flexibility under conditions of nutrient limitation or therapeutic stress [29,30]. Because malignant cells often exhibit increased dependence on metabolic plasticity, disruption of cellular energy homeostasis may preferentially affect cancer cells, compared with non-malignant cells [26,29].

In the present study, dose–response analysis demonstrated that when administered alone, QUE reduced MSTO-211H cell viability in a time-dependent manner, whereas ATG produced more moderate inhibitory effects. Importantly, concentrations selected for combination experiments maintained greater than 95% viability in MeT-5A cells, indicating minimal cytotoxic effects in non-malignant mesothelial cells. Differential sensitivity between malignant and non-malignant cells may reflect differences in mitochondrial regulation, metabolic demand, and stress adaptation capacity [29,30]. These characteristics are increasingly recognized as important determinants of cancer-selective susceptibility to metabolic stress-inducing agents.

Compared to single-treatment conditions, combination treatment produced greater reductions in mesothelioma cell viability, suggesting cooperative regulatory effects of QUE and ATG. Combination index analysis indicated synergistic effects in MSTO-211H cells, particularly at later time points, supporting the concept that bioactive polyphenols may exert complementary regulatory actions on multiple intracellular signaling pathways [31,32]. Polyphenolic compounds are known to influence diverse molecular targets, and combination approaches may enhance biological responses through the simultaneous modulation of distinct regulatory mechanisms, without requiring high concentrations of individual compounds [24,32]. Such combination strategies may therefore provide a means of enhancing biological effects while minimizing potential toxicity.

In addition to reduced cell viability, combined treatment significantly decreased intracellular ATP levels in mesothelioma cells, suggesting disruption of cellular energy homeostasis. Maintenance of ATP production is essential for cancer cell survival, particularly under conditions of metabolic stress associated with tumor progression [26,28]. Reduced ATP availability may activate adaptive signaling pathways that regulate cellular metabolism and stress responses [33,34]. The observed decrease in ATP levels following co-treatment suggests potential alteration of cellular bioenergetic regulation, and indicates that altered cellular energy balance may contribute to reduced proliferative capacity in mesothelioma cells.

Consistent with the observed reduction in cell viability, co-treatment significantly increased apoptotic cell populations, as demonstrated by Annexin V staining and increased caspase activity. Activation of caspase-dependent signaling pathways is a characteristic feature of programmed cell death, and reflects the coordinated regulation of intracellular proteolytic cascades [35,36]. Previous studies have shown that polyphenolic compounds may induce apoptosis through the modulation of mitochondrial membrane integrity and activation of caspase-associated signaling pathways [35,36,37]. The increased apoptotic cell populations observed in the present study indicate that combined treatment enhances the susceptibility of mesothelioma cells to apoptosis under the experimental conditions examined.

Western blot analysis further demonstrated alterations in the expression of mitochondrial apoptosis-associated proteins following co-treatment. Reduced expression of anti-apoptotic proteins Mcl-1, Bcl-2, and Bcl-xL was observed. Members of the Bcl-2 protein family play critical roles in regulating mitochondrial outer membrane permeability and cellular susceptibility to apoptosis [37]. Alterations in the balance between pro-apoptotic and anti-apoptotic proteins may promote mitochondrial outer membrane permeabilization and facilitate activation of downstream caspase signaling pathways [35,37]. Increased expression of cleaved caspase-3 and cleaved PARP further supports activation of apoptosis-associated proteolytic mechanisms. Cleavage of PARP is commonly considered a biochemical marker of apoptosis and reflects activation of caspase-mediated signaling processes [35,38]. These findings are consistent with increased caspase activity observed in enzymatic assays and support the interpretation that co-treatment promotes caspase-dependent apoptotic cell death.

In addition to apoptosis-related proteins, co-treatment influenced mitochondrial OXPHOS complex proteins and phosphorylation of AMP-activated protein kinase (AMPK). AMPK functions as a central regulator of cellular energy homeostasis and is activated in response to decreased ATP availability [33,39,40]. Activation of AMPK signaling pathways has been associated with suppression of anabolic processes and increased sensitivity to metabolic stress [39,40]. Increased phosphorylation of AMPK observed following co-treatment suggests activation of cellular stress response pathways associated with altered bioenergetic conditions. Alterations in OXPHOS complex protein expression further suggest changes in mitochondrial respiratory protein expression patterns under the experimental conditions tested. Mitochondrial oxidative phosphorylation represents a major source of ATP production in many cell types, and disruption of respiratory complex activity may reduce cellular bioenergetic capacity [41,42].

Collectively, these observations support the concept that combined treatment is associated with metabolic stress-related changes in mitochondrial regulatory pathways, which may contribute to activation of apoptosis signaling pathways in mesothelioma cells.

Targeting mitochondrial metabolism has been proposed as a potential strategy to selectively affect cancer cells exhibiting altered metabolic phenotypes [28,41,43].

**Figure 7:**
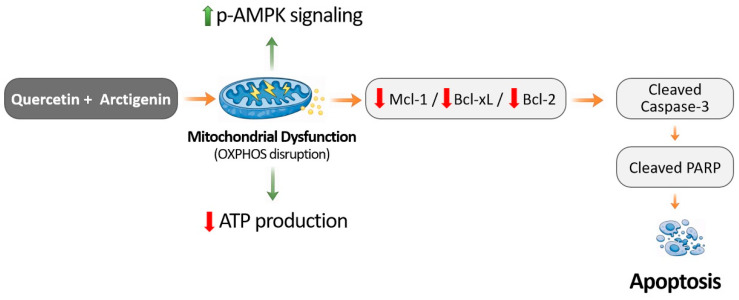
Proposed mechanism underlying the cooperative effects of quercetin and arctigenin in mesothelioma cells. Schematic illustration of the proposed mechanism by which co-treatment with quercetin (QUE) and arctigenin (ATG) reduces mesothelioma cell viability through induction of metabolic stress and apoptosis-associated signaling pathways. Combined treatment decreases intracellular ATP production and activates AMPK signaling, accompanied by altered mitochondrial OXPHOS protein expression patterns. These metabolic alterations are associated with reduced expression of anti-apoptotic proteins Mcl-1, Bcl-2, and Bcl-xL, leading to activation of caspase-dependent apoptotic signaling, as evidenced by increased cleaved caspase-3 and cleaved PARP, ultimately contributing to apoptotic cell death in MSTO-211H mesothelioma cells. Arrows indicate the direction of signaling interactions, while upward and downward arrows represent increases and decreases in protein expression or activity, respectively.

Figure 7 summarizes mechanistic relationships among ATP reduction, AMPK activation, mitochondrial signaling, and apoptosis induction. The proposed model illustrates how combined exposure to QUE and ATG may influence cellular energy metabolism and mitochondrial regulatory pathways, ultimately contributing to activation of apoptosis-associated signaling processes [39,41]. Modulation of mitochondrial regulatory processes and bioenergetic capacity may represent an important component of cooperative effects observed under co-treatment conditions. Integration of metabolic stress responses with apoptosis-associated signaling pathways may provide insight into mechanisms by which polyphenolic compounds influence cellular stress adaptation.

Importantly, molecular alterations observed in MSTO-211H cells were less pronounced in MeT-5A cells, supporting cancer-selective effects of the selected treatment conditions. Differential responses between malignant and non-malignant cells may reflect differences in mitochondrial dynamics, metabolic flexibility, and stress adaptation capacity [26,29,43]. Cancer cells often exhibit increased metabolic demand and may therefore be more susceptible to agents that influence mitochondrial-associated metabolic regulation and cellular energy homeostasis [28,41]. These observations are consistent with the concept that metabolic vulnerabilities may represent potential targets for therapeutic intervention.

Several limitations of the present study should be considered. Findings are based primarily on in vitro experimental models, and additional studies are required to determine whether similar effects occur in more complex biological systems. Further investigation of mitochondrial functional parameters, including respiration rate, mitochondrial membrane potential, and reactive oxygen species production, may provide additional mechanistic insight into cellular responses observed following co-treatment [29]. Evaluation of combination effects in 3D culture systems or in vivo models may further clarify the translational relevance of these findings [43]. Future studies examining interactions between metabolic stress signaling pathways and tumor microenvironment-associated factors may further improve understanding of the cooperative effects of bioactive compounds.

Taken together, the present findings suggest that combined treatment with QUE and ATG is associated with reduced mesothelioma cell viability and increased apoptotic cell death through modulation of mitochondrial apoptosis-associated signaling pathways. Reduced ATP levels, increased AMPK phosphorylation, altered expression of Bcl-2 family proteins, and activation of caspase-dependent signaling collectively suggest involvement of metabolic stress-associated regulatory mechanisms. These findings provide insight into potential cooperative effects of polyphenols on cellular stress responses and support further investigation of combination strategies targeting metabolic mechanisms in mesothelioma cells.

## 5. Conclusions

In the present study, combined treatment with quercetin and arctigenin was associated with greater inhibition of mesothelioma cell viability while maintaining minimal cytotoxicity in non-malignant mesothelial MeT-5A cells. Experimental concentrations were selected based on preservation of MeT-5A viability above 95%, allowing evaluation of relatively selective responses in malignant cells under conditions minimizing non-specific toxicity. Co-treatment reduced intracellular ATP levels and increased phosphorylation of AMPK, suggesting induction of cellular energy stress. These metabolic alterations were accompanied by increased apoptotic cell populations, elevated caspase activity, and enhanced expression of cleaved caspase-3 and cleaved PARP. In addition, decreased expression of anti-apoptotic proteins Mcl-1, Bcl-2, and Bcl-xL is consistent with potential involvement of mitochondrial apoptosis-associated regulatory pathways. Collectively, these findings suggest that combined exposure to QUE and ATG is associated with metabolic stress-related changes linked to apoptosis signaling in mesothelioma cells. This study provides insight into potential cooperative effects of polyphenols on mitochondrial regulatory pathways and supports further investigation of combination strategies targeting metabolic mechanisms in mesothelioma.

## Data Availability

The original contributions presented in the study are included in the article; further inquiries should be directed to the corresponding author.
